# An innovative oral management procedure to reduce postoperative complications

**DOI:** 10.1016/j.xjon.2022.01.021

**Published:** 2022-02-16

**Authors:** Akari Kaga, Tetsuya Ikeda, Keisei Tachibana, Ryota Tanaka, Haruhiko Kondo, Takanori Kawabata, Tomoko Yorozu, Koichiro Saito

**Affiliations:** a Department of Oral Surgery, Kyorin University School of Medicine, Tokyo, Japan; b Department of General Thoracic Surgery, Kyorin University School of Medicine, Tokyo, Japan; c Clinical Research Center, Shizuoka Cancer Center, Shizuoka, Japan; d Department of Anesthesiology, Kyorin University School of Medicine, Tokyo, Japan; e Department of Otorhinolaryngology, Kyorin University School of Medicine, Tokyo, Japan

**Keywords:** hospital stay period, lung cancer surgery, oral management, oral stimulation, postoperative complications, C-D, Clavian-Dindo, ECOG, Eastern Cooperative Oncology Group, PS, performance status

## Abstract

**Background:**

Numerous studies have shown that improving oral hygiene contributes to a reduction in the risk of postoperative complications in patients with head and neck cancer, cardiac disease, and esophageal cancer. However, the beneficial standard for oral management procedures during the perioperative period has not yet been established. Therefore, our aim was to determine whether or not their innovative oral management intervention contributed to a reduction in postoperative complications in lung cancer.

**Methods:**

We performed a retrospective analysis of medical records of patients who underwent lung cancer surgery with lobectomy and pneumonectomy at Kyorin University Hospital. Patients were divided into 2 groups: a perioperative oral management intervention group that underwent lung cancer surgery from April 2016 to March 2018 (n = 164), and a control group without oral management that underwent surgery from April 2014 to March 2016 (n = 199). In particular, our oral management procedure emphasized oral mucosa stimulation to induce saliva discharge as in gum chewing, rather than simply using teeth brushing to reduce oral microbiome. Therefore, our oral management procedure is different from traditional oral care.

**Results:**

This study demonstrated that our oral management practice was associated with a decline in the occurrence of postoperative pneumonia (odds ratio, 0.184; 95% CI, 0.042-0.571; *P* = .009), postoperative hospital stay duration (β coefficient, −4.272; 95% CI, −6.390 to −2.155; *P* < .001) and Clavian-Dindo classification grade II or above (odds ratio, 0.503; 95% CI, 0.298-0.835; *P* = .009).

**Conclusions:**

We propose an innovative new strategy using their unique oral management procedure to reduce postoperative complications resulting from pulmonary resection.

## Graphical abstract

**Figure:**
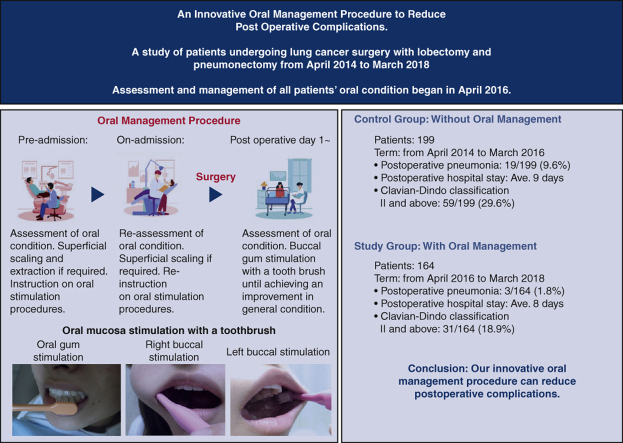

**Figure:**
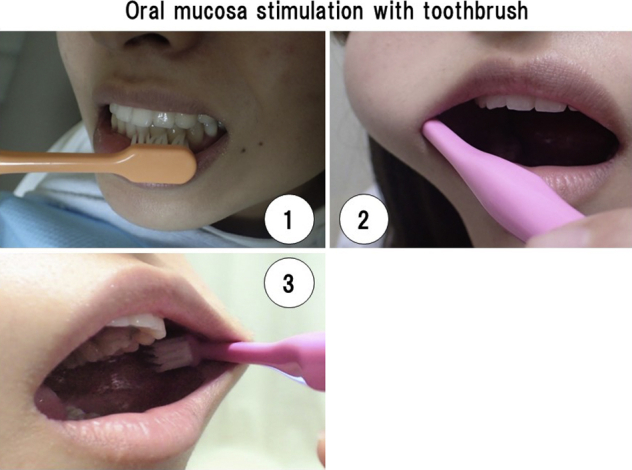
Oral mucosa stimulation with a toothbrush. 1, Gum. 2, Right buccal mucosa. 3, Left buccal mucosa.

**Central Message**

Our innovative oral management procedure is safe, efficient, cost-effective, and cogent; it could be applied in hospitals immediately.

**Perspective**

This study reveals that, in cases of pulmonary resection, our innovative oral management procedure has a propensity to decrease the development of postoperative pneumonia and Clavian-Dindo classification II or above when compared to the nonintervention group. Furthermore, our intervention leads to a decrease in postoperative hospital stay compared to the nonintervention group.

See Commentary on page 454.

Postoperative complications are associated with increased mortality, postoperative hospital stay, and cost.^1^^,^^2^ Numerous studies show that improving oral hygiene contributes to a reduction in the risk of postoperative complications in patients with cardiac disease^3^ and lung cancer.^4^ Furthermore, postoperative pneumonia in esophageal cancer patients was reduced from 32% to 9% by brushing their teeth 5 times a day commencing 1 week before the date of the operation.^5^ A study of patients undergoing cardiac surgery revealed those with unsatisfactory oral hygiene had 12 times the chance of developing postoperative pneumonia compared with patients with satisfactory oral hygiene.^6^ Iwata and colleagues^4^ reported that patients who received perioperative oral care from a dentist and a dental hygienist experienced fewer complications after lung resection, 13 of 280 patients (4.6%), than patients who received no perioperative oral care, 41 of 441 patients (9.3%). In addition, Postoperative pneumonia is still a common complication after major pulmonary resections, with an incidence of 5.0% to 10.9%.^7^^,^^8^ Therefore, reducing the frequency of postoperative pneumonia remains a problem.

Ishimaru and colleagues^9^ described that in patients with esophageal, gastric, and colon cancers there is a significant statistical relationship between oral care and postoperative complications. In contrast, there is no statistically significant relationship between oral care and postoperative pneumonia in patients with lung, liver, and head-neck cancers. Therefore, the relationship between preoperative oral care and postoperative complications may be greater in patients with gastrointestinal tract cancer than in patients with other types of cancer (eg, liver, lung, and head-neck cancer). Thus, these reports indicate that the beneficial standard for oral management procedures for all types of disease during the perioperative period has not yet been established.

Researchers estimate that the oral microbiome includes more than 500 bacterial species.^10^^,^^11^ Poor oral hygiene was among the risk factors contributing to nosocomial infection.^12^ Also, many microorganisms of tracheal aspirate cultures were concordant with dental plaque bacteria.^12^ Researchers recognized that the endotracheal tube served as a vehicle for provider microorganisms to the lower respiratory tract.^12^, ^13^, ^14^ Judging from those reports, improving oral hygiene before the operation would have significant benefits in patients who also had a surgical wound site on the lung.

Since 2015, Kyorin University Hospital anesthesiologists have examined all patients scheduled to undergo surgery under general anesthesia, before their surgery. In the same department, since April 2016, Kyorin University Hospital dental staff members have checked the oral condition of all patients who underwent surgery under general anesthesia, including primary malignant pulmonary tumor resection. The purpose of this study is to determine whether or not our new oral management intervention, mainly characterized by oral mucosa stimulation with a toothbrush, contributes to a reduction in postoperative pneumonia, hospital stay period, and postoperative complications resulting from pulmonary resection.

## Methods

### Patients

We performed a retrospective analysis of medical records of patients who underwent lung cancer surgery from April 2014 to March 2018 at Kyorin University Hospital. We extracted those patients who underwent lobectomy and pneumonectomy, leaving 363 patients in this study; 357 patients underwent lobectomy and 6 patients underwent pneumonectomy. The exclusion criteria were operative procedures on the other lung surgery, including wedge resection and segmentectomy, emergency surgery, palliative surgery, noncancer disease, and metastasis. We excluded 31 patients comprising 10 who rejected or received an incomplete oral intervention procedure, 2 with Eastern Cooperative Oncology Group (ECOG) performance status (PS) of 2 or greater, 11 with multifocal lung cancer, 4 with unknown histologic type, 2 with latent carcinoma, and 2 patients with uncertain postoperative complication history.

**Figure 1:**
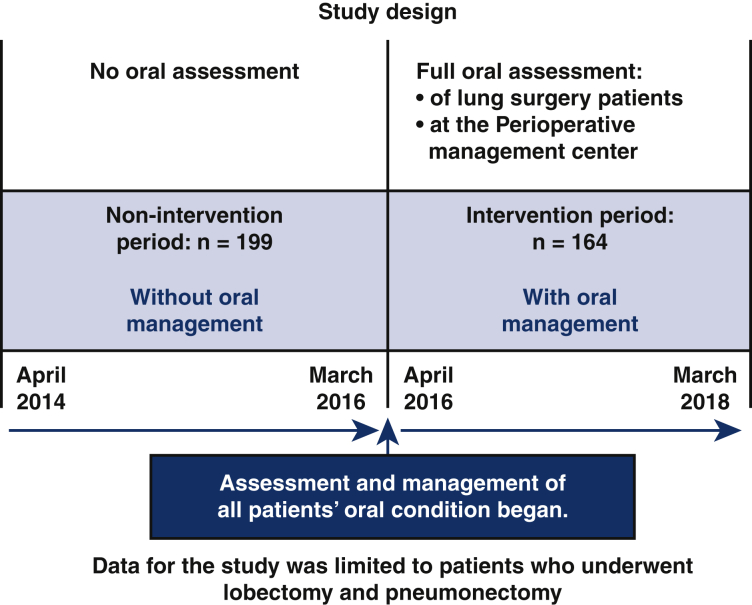
The 2 groups in this study. The intervention group had full oral assessment by dentists and dental hygienists at Kyorin University Hospital before general anesthesia lung cancer surgery between April 2016 and March 2018. The nonintervention group had no oral assessment and lung cancer surgery between April 2014 and March 2016. The study was limited to lung cancer patients who had lobectomy and pneumonectomy.

We divided the study's patients into 2 groups: an oral management intervention group that underwent lung cancer surgery from April 2016 to March 2018 and was assessed by a dentist and a dental hygienist during perioperative period (n = 164), and a control group, without oral management, that underwent lung cancer surgery from April 2014 to March 2016 (n = 199) (Figure 1). Our intervention period extended from 2 weeks before surgery to the time when patients could intake orally and discharge adequate salivary flow.

### Complication Diagnosis

We diagnosed pneumonia in accordance with reports from previous researchers.^15^ Furthermore, we defined and assessed postoperative complication according to Clavien-Dindo (C-D) classification.^16^^,^^17^

### Oral Management

**Figure 2:**
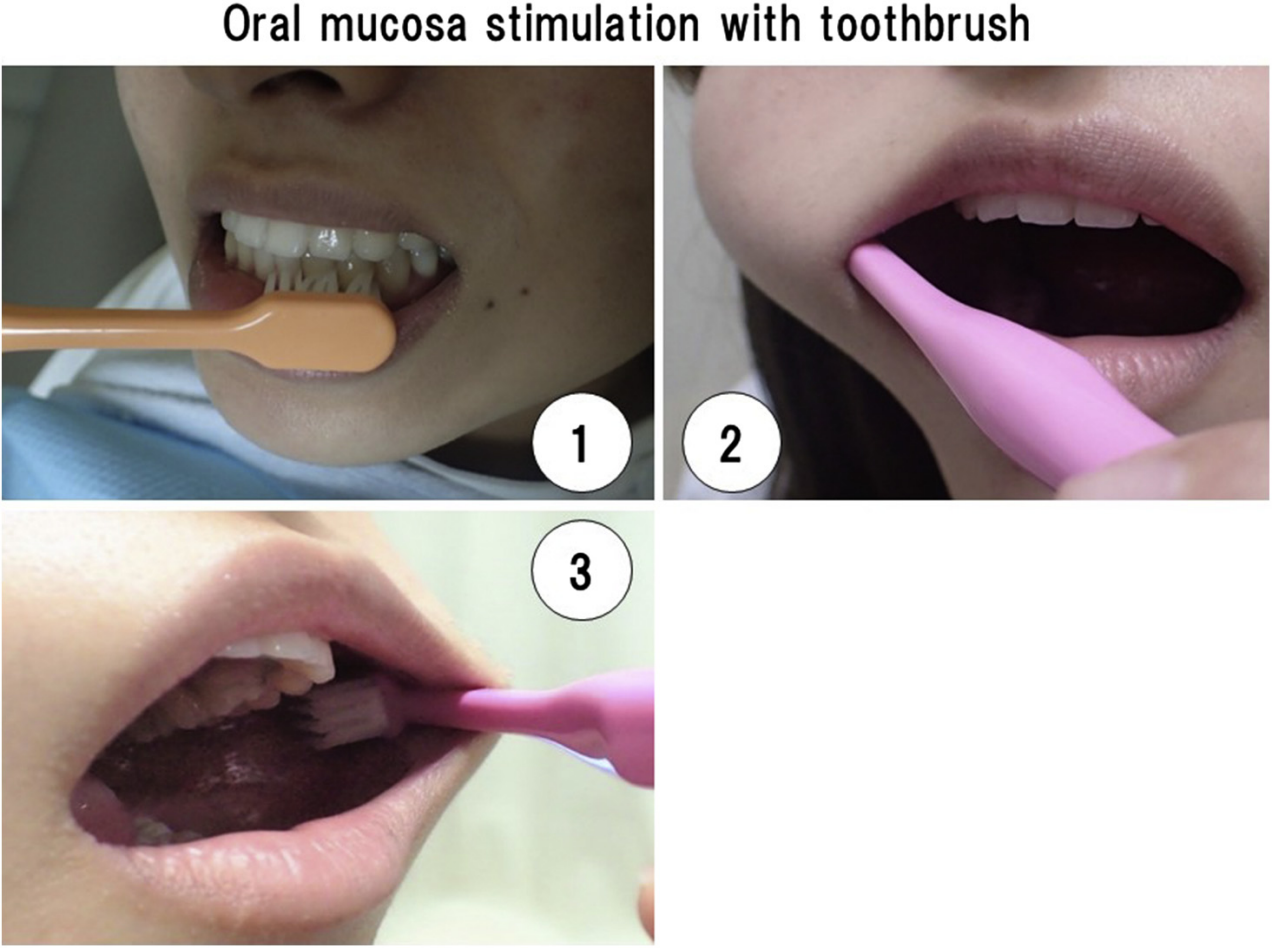
Oral mucosa stimulation with toothbrush. 1, Gum. 2, Right buccal mucosa. 3, Left buccal mucosa.

**Figure 3:**
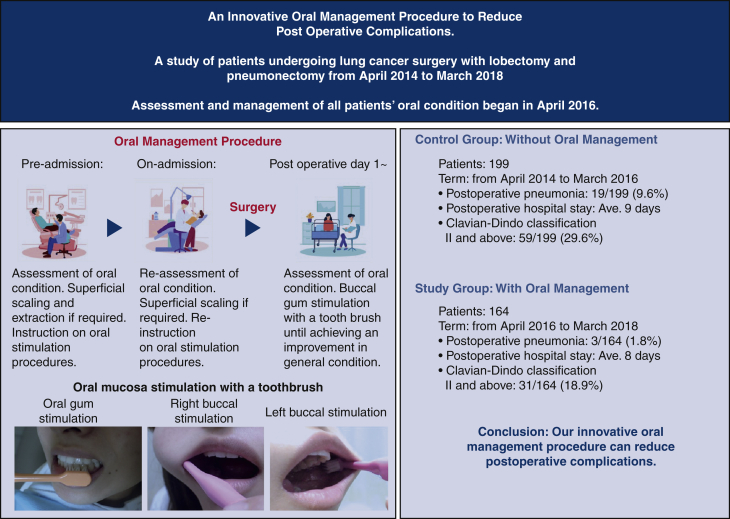
Subjects were divided into 2 groups. Patients with no oral management intervention who underwent surgery from April 2014 to March 2016 and patients with our oral management intervention who underwent surgery from April 2016 to March 2018. All patients from April 2016 had our oral management intervention. Patients were instructed on how to stimulate oral mucosa with a toothbrush. Our oral management intervention reduced the number of postoperative complications from pneumonia, hospital stay, and Clavian-Dindo classification Ⅱ and greater.

Before admission, we conducted common oral hygiene practice and instruction, such as dental scaling, removal of plaque with a toothbrush, cleaning of dentures, and use of an oral rinse, excluding a disinfectant agent. It also included dental extraction where there was severe periodontitis showing pain, pus discharge, and mobility before the operation. The purpose of this tooth extraction was to facilitate smooth intubation. As a result, 9 patients had a total of 12 teeth extracted. We emphasized that our instructions were to improve oral hygiene but more importantly for patients to stimulate oral mucosa by themselves with a toothbrush, as many times as possible (See Figures 2 and 3). This oral mucosa stimulation process is the foundation of our oral management procedure, and is clearly shown in the accompanying Video 1. We instructed patients to continue this procedure from the day of assessment of their oral condition at the perioperative management center, through the surgery period and afterward, until their general condition improved. Specifically, the time immediately before going to the operating theatre is especially important for this oral stimulation. This is because we believe oral mucosa stimulation acts as sham feeding that may induce bowel movement as in gum chewing.^18^ Because our oral stimulation with a toothbrush moved jaw muscles, this procedure may have induced saliva secretion. In contrast, we did not use some oral management practices widely accepted among health professionals. These included measurement of periodontal pocket depth, the use of oral rinse, including an oral antiseptic agent (eg, chlorhexidine gluconate), and cleaning of the tongue surface. Also, decayed teeth were sealed with filling materials. Therefore, our oral management procedure of oral mucosa stimulation with a toothbrush begins during the preoperative period and continues right through until there is an improvement in general condition after surgery (See Figure 3).

### Variables

We examined the following variables using the patients’ medical records: age, gender, body mass index, ECOG PS, vital capacity, forced expiratory volume for 1 second expressed as a percentage of the forced vital capacity, comorbidities (eg, artificial dialysis, interstitial pneumonia, ischemic cardiac disease, cranial nerve disease or cerebrovascular disorder, diabetes mellitus, autoimmune disorder, arrhythmia, and hypertension), smoking status, preoperative therapy, size of tumor, surgical side, albumin, operation time, blood loss, surgical approach, histologic type, pathologic staging, and postoperative outcomes, such as postoperative hospital stay duration, postoperative pneumonia, and C-D classification. In this study, we regarded patients with postoperative complications as having C-D classification grade II or above.

### Statistical Analysis

We assessed categorical variables with Fisher exact test and continuous variables using the Mann-Whitney *U* test. We performed negative binomial regression analysis to evaluate the association with postoperative hospital stay duration. We conducted logistic regression analysis for binary data such as the occurrence of postoperative pneumonia and C-D classification II or greater. We included variables with *P* < .05 on univariate analysis in the subsequent multivariate regression analysis and we applied this criterion for both negative binomial and logistics regression analysis. We performed all statistical analyses using R version 3.6.1 (R Foundation for Statistical Computing).

### Data Accuracy

In our study design, we clearly defined risks and outcomes and used objective methods. We addressed selection bias by accepting all patients who underwent anesthesia surgery in Kyorin Hospital in the period 2016 to 2018. There was no selection of patients. We used objective data sources, medical records, and objective diagnostic studies for statistical analysis. We engaged a large number of authors, with extensive research backgrounds, collaborating and cross-checking our study design, data sourcing, analysis, and conclusions to act as natural checks and balances.

The Institutional Ethics Committee of Kyorin University Hospital approved this study (No. 1192). The authors published a research plan and guaranteed an opt-out opportunity to our hospital according to the instructions of the institutional review board. The Institutional Ethics Committee of Kyorin University permitted us to use individual patient information because we did not identify any individual patients.

## Results

The study analyzed the background data of 363 patients in 2 groups. The oral management group comprised 164 patients with 114 (69.5%) men and 50 (30.5%) women with an average age of 69 years (range, 30-91 years). The control group comprised 199 patients with 123 (61.8%) men and 76 (38.2%) women with an average age of 69 years (range, 38-90 years). In addition, Tables 1 and 2 show that gender, age, body mass index, forced expiratory volume for 1 second expressed as a percentage of the forced vital capacity, comorbidities (eg, artificial dialysis, interstitial pneumonia, ischemic cardiac disease, cranial nerve disease or cerebrovascular disorder, diabetes mellitus, autoimmune disorder, and arrhythmia), smoking status, preoperative therapy, surgery side, surgical approach, and histologic type were evaluated and there were no significant differences between the 2 groups. In contrast, PS, vital capacity, hypertension, size of tumor, and albumin level were detected differences.

**Table 1:** Patient characteristics and surgical information

Characteristic	Oral management group (n = 164)	No oral management group (n = 199)	*P* value
Gender			
Male	114 (69.5)	123 (61.8)	.150
Female	50 (30.5)	76 (38.2)	
Age (y)	69 (30-91)	69 (38-90)	.768
Body mass index	22.75 (16.19-32.71)	22.75 (0.00-42.31)	.663
ECOG performance status			
0	155 (94.5)	171 (85.9)	.008∗
1	9 (5.5)	28 (14.1)	
%VC	103.40 (24.30-1306.00)	108.40 (42.50-153.50)	.012∗
%FEV1	73.30 (45.90-717.00)	74.20 (27.10-708.00)	.202
Comobidities			
Artificial dialysis	1 (0.6)	1 (0.5)	1.000
Interstitial pneumonia	2 (1.2)	3 (1.5)	1.000
Ischemic cardiac disease	4 (2.4)	13 (6.7)	.080
Cranial nerve disease or cerebrovascular disorder	4 (2.4)	11 (5.6)	.186
Diabetes mellitus	22 (13.4%)	29 (14.9)	.762
Autoimmune disorder	2 (1.2%)	1 (0.5)	.594
Arrhythmia (medical treatment)	6 (3.7)	6 (3.1)	.777
Hypertension	49 (29.9)	9 (4.6)	<.001∗
Smoking status			
Never smoked	42 (39.3)	65 (60.7)	.301
Quit ≥30 d before surgery/exsmoker	103 (47.0)	116 (53.0)	
Quit <30 d before surgery/still smoking	19 (51.4)	18 (48.6)	
Preoperative therapy	1 (0.6)	4 (2.0)	.383
Size of tumor (cm)	2.90 (0.90-9.20)	2.50 (0.80-9.10)	.001∗
Side			
Right	112 (68.3)	132 (66.3)	.737
Left	52 (31.7)	67 (33.7)	
Albumin (g/dL)	4.20 (2.60-5.00)	4.30 (2.50-5.20)	.017∗

Values are presented as median (min-max) or n (%). *ECOG*, Eastern Cooperative Oncology Group; *%VC*, vital capacity; *%FEV1*, forced expiratory volume for 1 second expressed as a percentage of the forced vital capacity.

∗ *P* < .05.

**Table 2:** Clinical outcome and surgical related information

Variable	Oral management group (n = 164)	No management group (n = 199)	*P* value
Operation time (min)	238.5 (99.0-598.0)	209.0 (90.0-515.0)	<.001∗
Blood loss (mL)	29.5 (0-6162)	39 (2-811)	.032∗
Surgical approach			
VATS	156 (95.1)	195 (98.0)	.149
Thoracotomy	8 (4.9)	4 (2.0)	
Histologic type			
Adenocarcinoma	112 (68.3)	145 (72.9)	.355
Nondenocarcinoma	52 (31.7)	54 (27.1)	
Pathologic staging			
0	4 (2.4)	6 (3.0)	<.001∗
IA and IB	70 (42.7)	122 (61.3)	
IIA and IIB	40 (24.4)	42 (21.1)	
IIIA and IIIB	37 (22.6)	28 (14.1)	
IV	13 (7.9)	1 (0.5)	
Clavian-Dindo classification	31 (18.9)	59 (29.6)	.020∗
Postoperative pneumonia	3 (1.8)	19 (9.6)	.002∗
Postoperative hospital stay	8 (2-113)	9 (4-89)	.029∗

Values are presented as median (min-max) or n (%).*VATS*, Video-assisted thoracoscopic surgery.

∗ *P* < .05.

Table 2 shows patients who underwent oral management had an average postoperative hospital stay of 8 days (range, 2-113 days) compared with 9 days for patients without oral management (range, 4-89 days; *P* = .029). There was also a significant difference in the occurrence of postoperative pneumonia between the oral management group, 3 of 164 patients (1.8%) and the control group, 19 of 199 patients (9.6%) (*P* = .002). This significant difference was also reflected in patients with a C-D classification of grade II or greater, 31 of 164 patients (18.9%) in the care group and 59 of 199 patients (29.6%) (*P* = .02) in the control group. Table 3 shows that oral management (odds ratio [OR], 0.184; 95% CI, 0.042-0.571; *P* = .009) was the only relative factor for postoperative pneumonia.

**Table 3:** Association with postoperative pneumonia (n = 363)

Variable∗	Univariate		Multivariate	
	Odds ratio (95% CI)	*P* value	Odds ratio (95% CI)	*P* value
Oral management				
No†	1	–	1	–
Yes	0.177 (0.041-0.530)	0.006‡	0.184 (0.042-0.571)	.009‡
Gender				
Male†	1	–		
Female	0.535 (0.172-1.390)	.230		
Age (y)	1.050 (0.998-1.110)	.074		
Body mass index	1.033 (0.918-1.155)	.583		
ECOG performance status				
0†	1	–	1	–
1	4.838 (1.734-12.459)	.001‡	2.883 (0.927-8.127)	.053
%VC	0.996 (0.972-1.004)	.738		
%FEV1	0.971 (0.935-1.002)	.130		
Comorbidities				
Artificial dialysis†	0.000 (0-∞)	.990		
Interstitial pneumonia†	0.000 (0-∞)	.990		
Ischemic cardiac disease	2.267 (0.342-8.849)	.300		
Cranial nerve disease or cerebrovascular disorder	1.157 (0.062-6.221)	.891		
Diabetes mellitus	1.984 (0.625-5.345)	.202		
Autoimmune disorder	8.400 (0.381-91.360)	.088		
Arrhythmia (medical treatment)	1.486 (0.079-8.239)	.711		
Hypertension	0.530 (0.083-1.896)	.402		
Smoking status				
Never smoked	1 (ref)	–		
Quit ≥30 d before surgery/exsmoker	2.030 (0.723-7.222)	.216		
Quit <30 d before surgery/still smoking	1.471 (0.198-7.881)	.664		
Preoperative therapy†				
No	1 (ref)	–		
Yes	0.000 (-∞)	.990		
Size of tumor (cm)	1.148 (0.868-1.466)	.297		
Surgical side				
Right†	1	–		
Left	0.586 (0.189-1.523)	.305		
Operation time (min)	1.002 (0.996-1.007)	.577		
Blood loss (mL)	1.000 (0.998-1.001)	.799		
Surgical approach§				
Thoracotomy†	1	–		
VATS	2.845E+6 (0-)	.990		
Histologic type				
Adenocarcinoma†	1	–	1	–
Nonadenocarcinoma	2.589 (1.075-6.242)	.032‡	2.221 (0.852-5.708)	.096
Pathologic staging§				
0†	1	–		
IA and IB	1.610E+6 (0-)	.991		
IIA and IIB	5.245E+6 (0-)	.990		
IIIA and IIIB	3.545E+6 (0-)	.990		
IV	3.273E+6 (0-)	.990		
Albumin (g/dL)	0.397 (0.175-0.951)	.030‡	0.562 (0.231-1.404)	.208

*ECOG*, Eastern Cooperative Oncology Group; *%VC*, vital capacity; *%FEV1*, forced expiratory volume for 1 second expressed as a percentage of the forced vital capacity; *VATS*, video-assisted thoracoscopic surgery.

∗ Number of events = 22.

† Reference category.

‡ *P* < .05.

§ An extremely large or near-zero odds ratio is due to the small number of postoperative pneumonia events (known as the complete separation).

Table 4 describes the associations between patient characteristics and the length of postoperative hospital stay. The negative binomial regression analysis found 5 predictor factors that were also associated with the length of postoperative hospital stay in this study: oral management (β coefficient, –4.272; 95% CI, –6.390 to –2.155; *P* < .001), PS (β coefficient, 1.246; 95% CI, 1.033-1.507; *P* = .022), size of tumor (β coefficient, 0.856; 95% CI, 0.096-1.617; *P* = .027), operation time (β coefficient, 0.030; 95% CI, 0.013-0.046; *P* < .001), blood loss (β coefficient, 0.004; 95% CI, 0.001-0.007; *P* = .008), surgical approach (β coefficient, –11.173; 95% CI, –17.263 to –5.082; *P* < .001), and albumin level (β coefficient, 0.847; 95% CI, 0.740-0.967; *P* = .014).

**Table 4:** Association with postoperative hospital stay (n = 363)

Variable	Univariate		Multivariate	
	Relative risk (95% CI)	*P* value	Relative risk (95% CI)	*P* value
Oral management				
No∗	1		1	
Yes	0.810 (0.712-0.920)	.001†	0.725 (0.645-0.815)	<.001∗
Gender				
Male∗	1	–	1	–
Female	0.810 (0.708-0.928)	.002†	1.063 (0.921-1.228)	.401
Age (y)	1.004 (0.997-1.011)	.257		
Body mass index	0.996 (0.979-1.014)	.644		
ECOG performance status				
0∗	1	–	1	–
1	1.416 (1.159-1.743)	.001†	1.246 (1.033-1.507)	.022†
%VC	0.999 (0.998-1.0002)	.148		
%FEV1	0.999 (0.998-1.001)	.288		
Comorbidities				
Artificial dialysis	1.097 (0.493-2.831)	.833		
Interstitial pneumonia	0.961 (0.566-1.725)	.888		
Ischemic cardiac disease	1.070 (0.797-1.460)	.659		
Cranial nerve disease or cerebrovascular disorder	1.334 (0.984-1.844)	.071		
Diabetes mellitus	1.193 (0.997-1.434)	.057		
Autoimmune disorder	1.211 (0.630-2.575)	.589		
Arrhythmia (medical treatment)	1.225 (0.871-1.765)	.258		
Hypertension	1.001 (0.842-1.195)	.993		
Smoking status				
Never smoked∗	1	–	1	–
Quit ≥30 d before surgery/exsmoker	1.486 (1.288-1.714)	.001†	1.241 (1.059-1.454)	.007†
Quit <30 d before surgery/still smoking	1.373 (1.096-1.729)	.006†	1.094 (0.867-1.378)	.436
Preoperative therapy				
No∗	1	–		
Yes	0.737 (0.425-1.343)	.295		
Size of tumor (cm)	1.123 (1.078-1.171)	<.001†	1.053 (1.010-1.099)	.013†
Surgical side				
Right∗	1	–		
Left	0.880 (0.768, 1.010)	.069		
Operation time (min)	1.0026 (1.0018-1.0033)	<.001†	1.002 (1.001-1.003)	<.001†
Blood loss (mL)	1.0007 (1.0004-0.0010)	<.001†	1.0002 (1.0001-1.0004)	.009†
Surgical approach				
Thoracotomy∗	1	–	1	–
VATS	0.456 (0.324-0.625)	<.001†	0.700 (0.502-0.958)	.025†
Histologic type				
Adenocarcinoma∗	1	–	1	–
Nonadenocarcinoma	1.313 (1.145-1.508)	<.001†	1.037 (0.909-1.183)	.589
Pathologic staging				
0∗	1	–	1	–
IA and IB	1.135 (0.758-1.667)	.529	0.953 (0.667-1.351)	.789
IIA and IIB	1.573 (1.040-2.337)	.028†	1.158 (0.797-1.669)	.434
IIIA and IIIB	1.827 (1.203-2.728)	.004†	1.125 (0.765-1.642)	.542
IV	1.080 (0.652-1.777)	.764	0.812 (0.740-0.967)	.377
Albumin (g/dL)	0.675 (0.589-0.775)	<.001†	0.847 (0.740-0.967)	.014†

*ECOG*, Eastern Cooperative Oncology Group; *VC*, vital capacity; *%FEV1*, forced expiratory volume for 1 second expressed as a percentage of the forced vital capacity; *VATS*, video-assisted thoracoscopic surgery.

∗ Reference category.

† *P* < .05.

Finally, Table 5 shows the factors contributing to a decrease in the development of the postoperative complication C-D classification II or greater. There was significant association with oral management (OR, 0.503; 95% CI, 0.298-0.835; *P* = .009), exsmoker (quitting smoking 30 days or more before surgery) (OR, 2.153; 95% CI, 1.192-4.057; *P* = .014), and blood loss (OR, 1.003; 95% CI, 1.001-1.005; *P* = .025). The exsmoker group had a propensity to postoperative complications. In contrast, the oral management intervention group had the propensity to reduced postoperative complications.

**Table 5:** Association of postoperative complications with Clavian-Dindo classification (n = 363)

Variable∗	Univariate		Multivariate	
	Odds ratio (95% CI)	*P* value	Odds ratio (95% CI)	*P* value
Oral management				
No†	1		1	
Yes	0.553 (0.334-0.902)	.019‡	0.503 (0.298-0.835)	.009‡
Gender				
Male†	1	–		
Female	0.753 (0.445-1.249)	.280		
Age (y)	1.024 (0.998-1.053)	.080		
Body mass index	0.959 (0.894-1.025)	.224		
ECOG performance status				
0†	1	–		
1	1.752 (0.830-3.556)	.128		
%VC	0.999 (0.991-1.003)	.788		
%FEV1	1.001 (0.995-1.005)	.766		
Comorbidities				
Artificial dialysis	3.103 (0.122-79.033)	.425		
Interstitial pneumonia	0.767 (0.039-5.272)	.814		
Ischemic cardiac disease	1.300 (0.404-3.618)	.631		
Cranial nerve disease or cerebrovascular disorder	1.126 (0.306-3.389)	.843		
Diabetes mellitus	1.498 (0.768-2.825)	.221		
Autoimmune disorder	6.279 (0.595-136.093)	.136		
Arrhythmia (medical treatment)	1.027 (0.224-3.534)	.968		
Hypertension	1.343 (0.704-2.475)	.355		
Smoking status				
Never smoked†	1	–	1	–
Quit ≥30 d before surgery/exsmoker	2.235 (1.258-4.150)	.008‡	2.153 (1.192-4.057)	.014‡
Quit <30 d before surgery/still smoking	1.460 (0.547-3.655)	.429	1.370 (0.497-3.537)	.526
Preoperative therapy				
No†	1			
Yes	0 (-,∞)	.982		
Size of tumor (cm)	1.070 (0.911, 1.249)	.398		
Surgical side				
Right†	1	–		
Left	0.843 (0.498-1.401)	.517		
Operation time (min)	1.002 (0.999-1.005)	.134		
Blood loss (mL)	1.003 (1.001-1.005)	.011‡	1.003 (1.001, 1.005)	.025‡
Surgical approach				
Thoracotomy†	1	–		
VATS	1.673 (0.431-11.020)	.512		
Histologic type				
Adenocarcinoma†	1	–		
Nonadenocarcinoma	1.210 (0.717-2.013)	.468		
Pathologic staging				
0†	1	–		
IA and IB	1.053 (0.252-7.156)	.950		
IIA and IIB	2.189 (0.507-15.144)	.342		
IIIA.and.IIIB	1.532 (0.343-10.796)	.611		
IV	0.308 (0.013-3.722)	.366		
Albumin (g/dL)	0.630 (0.370-1.076)	.087		

*ECOG*, Eastern Cooperative Oncology Group; *%VC*, vital capacity; *%FEV1*, forced expiratory volume for 1 second expressed as a percentage of the forced vital capacity; *VATS*, video-assisted thoracoscopic surgery.

∗ Number of events = 90.

† Reference category.

‡ *P* < .05.

## Discussion

This study aimed to investigate whether or not oral management intervention contributes to decreased postoperative complications in cases of pulmonary resection. Oral management before the operation seems to have a positive effect on the development of postoperative complications. Anatomically, the location of the trachea, the lungs, and the oral cavity may have a cause-and-effect relationship with these postoperative complications. The oral cavity and the trachea are connected by tracheal intubation. In particular, patients who underwent pulmonary resection have difficulty in coughing up sputum, a decline in respiratory function, an increase in sputum, and wound pain after surgery. These situations may have promoted postoperative complications. Therefore, oral management could be an important factor in decreasing postoperative complications for patients undergoing pulmonary surgery.

This study shows that, in cases of pulmonary resection, there has been a decline in the development of postoperative complications within a 4-year period. Although the advance of minimally invasive operations, such as video-assisted thoracic surgery, has had a positive effect on the prevention of postoperative complications, this study reveals that a decrease in postoperative hospital stay is not strongly linked to the operation method. Tables 3 and 5 show that there is no relationship between the operation method and postoperative pneumonia or C-D classification. However, this study also reveals that in cases of pulmonary resection, our extremely innovative oral management procedure leads to a decrease in the development of postoperative pneumonia, C-D classification II or greater, and postoperative hospital stay when compared with the nonintervention group.

ECOG PS 0 means a patient can work at a normal level of performance. PS 1 means the patient can perform tasks involved in ordinary living, but can not complete strenuous, aggressive work. Therefore a patient with PS 0 is better off than a patient with PS 1. However, making the distinction between PS 0 or PS 1 is difficult because it is a subjective evaluation. Because our data had a statistically significant result during the postoperative hospital stay period, PS was detected as a relevant confounding factor. In contrast, PS was not recognized as a relevant confounding factor in postoperative pneumonia and postoperative complications. Thus, this factor may have a relationship with recovery from general anesthesia damage (Table 4), but it might not be an important related factor for postoperative complications (Tables 3 and 5).

Yoneyama and colleagues^19^ demonstrated that there was no difference in pneumonia mortality rate between dentulous and edentulous elderly patients who had undertaken oral care. This indicated that aspiration pneumonia developed regardless of whether periodontally compromised teeth were present or not. Therefore, we believe perioperative oral management should be separated from periodontal treatment. Our oral management procedure is different from traditional oral care in its ability to reduce postoperative complications. The term traditional oral care usually refers to oral hygiene instruction, measurement of periodontal pocket depth, removal of dental calculus (ie, scaling), professional mechanical tooth cleaning, and removal of tongue coating with a toothbrush.^20^ In contrast, we have not adopted the use of oral rinse, including oral antiseptic such as chlorhexidine, because a previous report indicated that irrigating the oropharynx with chlorhexidine gluconate in lung cancer surgery was not effective in decreasing postoperative complication.^21^ On the other hand, Bardia and colleagues^22^ showed that preoperative chlorhexidine use is associated with a reduction in postoperative pneumonia after cardiac surgery. Nonetheless, this study reported no significant difference in duration of mechanical ventilation, length of intensive care unit stay, and length of hospital stay between the group of patients where chlorhexidine was used and the group where it was not used. In addition, the generalizability to patients undergoing surgeries other than cardiac is still unclear. These reports suggests the role of preoperative chlorhexidine mouthwash in preventing postoperative complications remains unclear. Therefore, we have not adopted the use of chlorhexidine. Funahara and colleagues^20^ described that the microorganisms of the mouth were reduced through perioperative oral care with their number immediately growing again the day after surgery, and further, that the number of microorganisms on the tongue after surgery differed between patients with intra oral intake and patients undergoing fasting. We speculate that the outcome was caused by the uncontrollability of the autonomic nervous system in connection with operative and general anesthesia stress. We believe that the microorganisms of the mouth increase with the decline of saliva secretion under conditions of sympathetic nerve advantage immediately after surgery. Further, we surmise that the cause of this outcome was the difference in levels of salivation between them for reasons including the lack of stimulus in fasted patients. In fact, Ryu and colleagues^23^ demonstrated that a decline in saliva secretion was associated with the growth of oral microorganisms, indicating a strong correlation between the amount of saliva and the number of salivary microorganisms. Therefore, because the postoperative oral condition is usually poor with a lack of saliva, it is so much more important and effective to promote saliva secretion with oral gum and mucosa stimulation with a toothbrush rather than removing tongue coating and/or using chlorhexidine mouthwash around the perioperative period. Previous reports indicate that gum chewing has been shown to direct vagal afferent stimulation of smooth muscle fibers and to stimulate secretion from salivary glands.^24^, ^25^, ^26^ These inputs can induce the inhibitory effects of sympathetic afferent pathways.^27^ Experience tells us that stimulation of oral mucosa and jaw muscles with a toothbrush induces vigorous salivary secretion, this phenomenon being under the regulatory control of the cerebral cortical masticatory area.^28^ Oral mucosa and jaw muscle stimulation with a toothbrush may have an effect similar to gum chewing. Thus, toothbrush stimulation may promote the secretion of saliva and stimulate the parasympathetic nervous system. We observed a trend toward fewer hospital stay days with our oral mucosa and jaw muscle stimulation, as a result of its innocuous nature, safe method, and low cost. We believe that the main objective of postoperative oral assessment should be to check whether or not patients can successfully intake orally and discharge adequate salivary flow. Therefore, our oral management procedure not only prevents bacterial infection but may also assist perioperative patients with recovery from general anesthesia. However, it is natural that an oral management protocol does not have the ability to cope with an unexpected situation. In this study, blood loss is valid. This oral stimulation scheme resulted in a statistically strong difference, but also huge ranges of blood loss with as much as >6000 mL in the oral management group. It is likely that the oral management protocol doesn't truly affect blood loss but may reflect confounding surgical factors that were not anticipated.

An interesting report described that there is a close correlation between the salivary and bronchoalveolar microbial compositions of patients with a greater salivary amylase activity level in bronchoalveolar lavage fluid.^29^ Lower levels of airway bacterial microbiome from the oral cavity may influence lung cancer recurrence after resection. Therefore, we believed that it was important to decrease oral microbiomes with sufficient amounts of saliva secretion in patients after lung surgery.

Many new efforts to reduce postoperative complications have been launched in recent years. The review studies regarding the effect of Enhanced Recovery After Thoracic Surgery on specific postoperative pulmonary complications, cost, and patient-reported outcomes such as pain scores and patient satisfaction scores are also warranted.^30^ In addition, the use of a comprehensive swallowing evaluation to detect aspiration before initiation of oral feedings after esophagectomy significantly reduced the incidence of pneumonia. This routine swallowing evaluation, incorporated into the postoperative care protocol after esophagectomy, demonstrated that postoperative pneumonia strongly predicted mortality.^31^ Since April 2016, our routine oral assessment was incorporated into a perioperative management system for all patients who undergo surgery requiring general anesthesia. It is very important to create and introduce systems that prevent patients’ postoperative complications before they occur.

This study has some limitations. It may indicate that preoperative oral intervention is connected to the occurrence of postoperative complications in general. However, because this was a retrospective study with a small size population in a single institution there is a possibility of unmeasured and unknown confounding factors.

No data were obtained from several confounders, including levels of smoking and oral condition. In particular, Table 3 shows that oral management was only 1 relative factor for postoperative pneumonia. But, if the number of participants increases, it is possible that other relative factors may arise, including PS and histologic type.

Table 5 shows the factors contributing to a decrease in the development of postoperative complication C-D classification II or greater. There was significantly more association with the exsmoker group, who quit smoking 30 days or more before surgery than with the group currently smoking. This means we could not verify actual smoking status from clinical records.

This study may not be generalizable to other countries because all of its participants were residents of Japan, a country with a universal public health insurance scheme providing all residents with medical care services. This system is quite different from health insurance in other countries. Further study is required, where a traditional oral care group is compared with our innovative oral management group using oral mucosa stimulation.

## Conclusions

This study reveals that, in cases of pulmonary resection, our extremely innovative oral management procedure can reduce postoperative pneumonia and C-D classification II or above when compared with the nonintervention group. Furthermore, our oral intervention leads to a decrease in postoperative hospital stay compared with the nonintervention group. This report proposes an innovative, simple, and efficient oral management procedure for the reduction of postoperative complications.

### Conflict of Interest Statement

The authors reported no conflicts of interest.

The *Journal* policy requires editors and reviewers to disclose conflicts of interest and to decline handling or reviewing manuscripts for which they may have a conflict of interest. The editors and reviewers of this article have no conflicts of interest.

## Supplementary Data

Video 1 This video demonstrates that our oral management procedure is different from traditional oral care because it is based on oral mucosa stimulation with a toothbrush to reduce postoperative complications. Video available at: https://www.jtcvs.org/article/S2666-2736(22)00061-4/fulltext.
