# Supplementary figures and images for: An innovative oral management procedure to reduce postoperative complications
Source: JTCVS Open. 2022 Feb 16;10:442–53. doi: 10.1016/j.xjon.2022.01.021 (PMC9390213; doi:10.1016/j.xjon.2022.01.021)

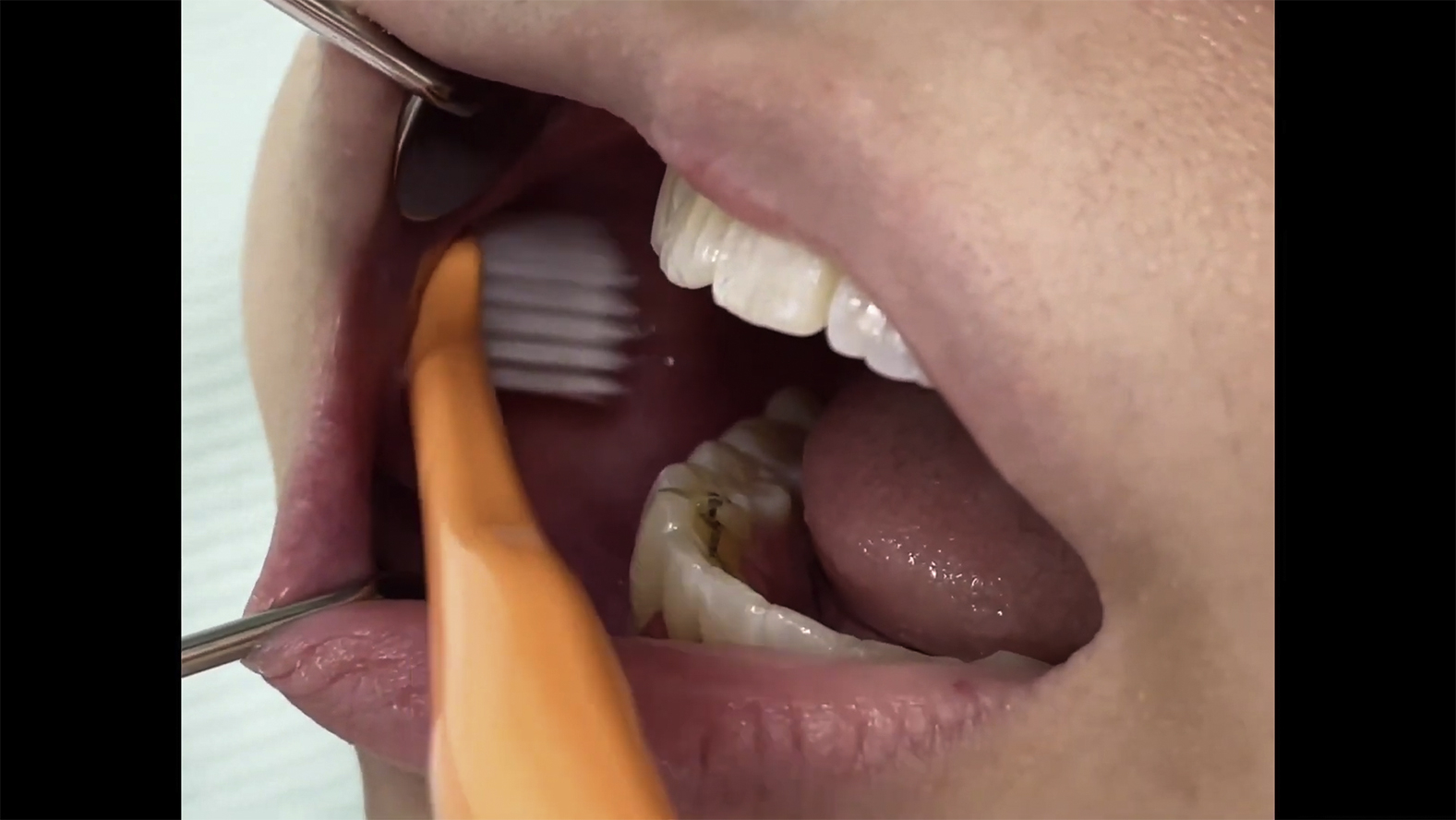

Supplement: Video 1 — This video demonstrates that our oral management procedure is different from traditional oral care because it is based on oral mucosa stimulation with a toothbrush to reduce postoperative complications. Video available at: https://www.jtcvs.org/article/S2666-2736(22)00061-4/fulltext. [file fx3.jpg]
